# Morphometric affinities and direct radiocarbon dating of the Toca dos Coqueiros’ skull (Serra da Capivara, Brazil)

**DOI:** 10.1038/s41598-022-11893-3

**Published:** 2022-05-12

**Authors:** Lumila Paula Menéndez, María Clara López-Sosa, Sergio Francisco Serafim Monteiro da Silva, Gabriela Martin, Anne-Marie Pessis, Niède Guidon, Ana Solari

**Affiliations:** 1 Department of Anthropology of the Americas, University of Bonn, Bonn, Germany; 2 Department of Evolutionary Biology, University of Vienna, Vienna, Austria; 3 Departamento de Arqueologia, Universidade Federal de Pernambuco, Recife, Brazil; 4 FUMDHAM Fundação Museu do Homem Americano, São Raimundo Nonato, Brazil; 5 INCT-INAPAS, Instituto Nacional de Ciencia e Tecnologia de Arqueologia, Paleontologia e Ambiente do Semiárido do Nordeste do Brasil, São Raimundo Nonato, Brazil

**Keywords:** Archaeology, Biological anthropology

## Abstract

The biological variation of the earliest skeletons of South America has been intensely debated for the last two centuries. One of the major research constraints has been the limited number of available samples dating to the early Holocene. We here present the first direct radiocarbon-date for the early Holocene human skeleton from Toca dos Coqueiros (Serra da Capivara, Brazil), also known as “Zuzu” (8640 ± 30 BP; 9526–9681 cal years BP). We performed craniometric analyses using exclusively samples from Brazil, to revisit the sex of the skeleton, and to discuss the evolutionary processes involved in the occupation of the continent. The sex of the individual was estimated as a female when compared to late and early Holocene individuals, but as a male when compared only to the early Holocene series. We also found that Zuzu presents the strongest differences with the late Holocene Guajajara individuals, located nearby, and the strongest similarities with the early Holocene series from Lagoa Santa, attesting for solid biological affinities among early Holocene individuals from Brazil, as well as a moderate level of morphological variation among them. This suggests that the early individuals were part of the same heterogeneous lineage, possibly a different one from which late Holocene populations diverged.

## Introduction

The debate surrounding the evolutionary processes shaping the diversification of the earliest Native American inhabitants has been ongoing for almost 200 years now^1–3^. Significant advances in the last few decades have been achieved thanks to several interdisciplinary attempts that provided comprehensive explanations^4–10^, the application of cutting-edge methods that allowed the capture of a larger amount of morphological information^11–16^, and the extraction, amplification, and sequencing of aDNA^17^. Some aspects of the expanding evolutionary process, such as the magnitude of biological variation that characterized the earliest inhabitants have become a relevant matter of discussion, especially in relation to the South American archaeological record^2,7,11–15,18–21^. While some authors have characterized the first Americans arriving in the continent as highly morphologically heterogeneous (i.e., large differences among individuals within a population), others described them as being mostly homogeneous (i.e., strong similarities among individuals)^16,18,20–23^. Understanding the degree of variation amongst these groups/individuals has implications for the models that can be proposed since it provides clues of the population size of the first migrants and/or the number of consecutive migrations by which humans arrived in the continent^11,18,19,21–23^. Another unanswered question in relation to the biological diversity of humans in the continent is whether the morphological changes that have been described across time represent different ancestral lineages outside America or are the result of local evolution^1,2,5,7,12–16,18–20^.

**Figure 1:**
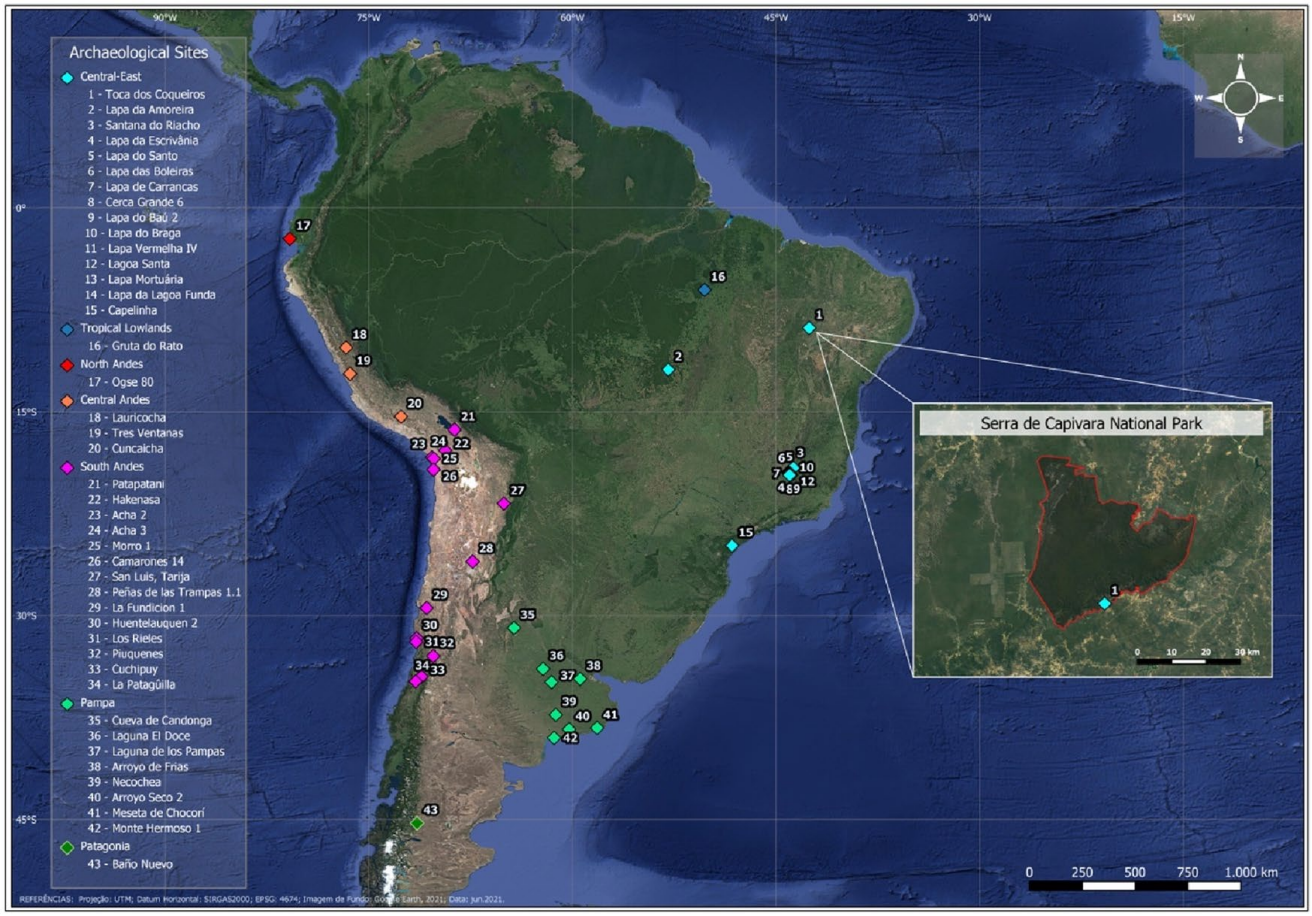
Archaeological sites from South America with direct early Holocene radiocarbon dates on human skeletons. The detailed figure shows the location of Toca dos Coqueiros site in the Serra da Capivara National Park. Color references indicate regions within South America. See Table S1 for a full list and information on the sites. Figure generated by extracting a map with UTM projection from Google Earth (https://earth.google.com/web/) and adding the location of the archaeological sites by using geographic coordinates with QGIS v.3.22.5 LTR (https://qgis.org/en/site/).

One of the major research constraints for advancing further in these inquiries has been the limited number of available samples dating to the early Holocene. As a result, most projects have been carried out by analyzing the few samples that are available, well preserved, and present radiocarbon dates that are accepted by a large part of the archaeological community. Currently, a total of 118 direct radiocarbon dates on human bones have been reported for the early Holocene in South America^24^. Most of these samples come from North, Central, and South Andes, as well as Patagonia, Pampas, Central-East South America (CESA), and fewer from the Tropical/Lowlands (see Table S1). For instance, the biological variation from the early Holocene individuals of CESA, which is represented by today’s Brazilian territory (including the South, Central-East, and North-East of Brazil), has been evaluated by studying mostly the Lagoa Santa skeletal series (7,500–11,500 ^14^C years BP)^25–27^, and to a lesser degree, the late Holocene Botocudo series from Central-East Brazil, which has been described as presenting similar craniometric variation to the early Holocene individuals^12,15,26,28–31^. The first skeletons from the Lagoa Santa skeletal series were recovered by Peter Lund in the 1830s and immediately became a relevant collection not only for South America but also at a worldwide level. The reasons behind this are that these individuals were recognized as a distinctive ancient human group (i.e., different to the contemporaneous Native Americans) that cohabited with the extinct Pleistocene megafauna^32,33^. Excavations and studies on this collection during the twentieth century were led especially by the Minas Gerais Academy of Sciences, the National Museum of Brazil, the French Mission, and Walter Neves’ team, and are continued today by their successorss^18,28,31,34–36^. However, there are also other archaeological sites in Brazil containing human remains associated with reliable radiocarbon dating (Fig. 1; for fully updated lists see^24,37^), that unfortunately have not been part of macroregional morphometric comparisons so far (but see for Santana do Riacho^38^).

The archaeological locality Serra da Capivara, situated in the state of Piaui, Brazil, presents, together with Lagoa Santa, the largest number of early Holocene archaeological sites that include human burials in Brazil^39–41^. Serra da Capivara has been intensively studied since the 1970s, when one of the current authors (NG) and her team started exploring the area^42–44^. Similar to Lagoa Santa, this area has played a key role in the debate surrounding the peopling of the Americas. Despite the debate concerning the very old age of some of the archaeological sites from Serra da Capivara, they contributed to the proposition of an earlier date for the peopling of the continent than the one that was accepted at the time, i.e., before 12,000 years BP^45–48^. The skepticism of some scholars in relation to the dating of the Serra de Capivara sites impacted negatively on the South American archaeological agenda, as it restrained other researchers from considering further the archaeological locality Serra da Capivara in reviews and debates on the earliest archaeological sites from the continent. Among the reported concerns, two can be highlighted: firstly, the extremely early dates that were proposed for some archaeological materials (e.g., lithic artifacts) based on associations with charcoal and/or the sediments containing them, i.e., 30,000–50,000 years^44,49,50^, and secondly, the absence of direct radiocarbon dates on the human bones^39,48^. Overall, this situation contributed to the dismissal of the sites from this locality, leaving them out of the South American archaeological agenda for many decades. However, thanks to recent morphometric studies and comprehensive reviews the Serra da Capivara skeletons are regaining a place in worldwide investigations, despite the continued absence of direct radiocarbon dating^39,48,51^.

The aim of this study was to analyze the morphology of the first individual from Serra da Capivara for which it was possible to extract dental enamel carbonate for radiocarbon determination by Accelerator Mass Spectrometry Method (AMS): the burial 1 from Toca dos Coqueiros, which includes an individual that is also known as “Zuzu”. Based on different comparative analyses, we present the results from three aspects that were analyzed for this skeleton: (1) chronological assessment (direct radiocarbon dating), (2) sex estimation, and (3) craniometric affinities in the context of the Brazilian/Central-Eastern South American early Holocene archaeological record.

For several decades attempts at obtaining direct radiocarbon dates from human bones or teeth of individuals from Serra da Capivara have failed, due to the lack of collagen. Until now, the only dates available have been those of charcoal associated with human remains (e.g., Toca da Janela da Barra de Antonião), sediment containing human remains (e.g., Toca do Paraguaio), or acid washes from the pretreatment of teeth (i.e., Toca do Garrincho), which are all indirect dates^39^. Here, we compare our new direct date with the current distribution of direct early Holocene radiocarbon dates on human bones and teeth from South America (North, Central, South Andes, Pampas, Patagonia), as well as with the most reliable direct dates for the Central-East of the continent, where this site is located (i.e., Central-East, North-East, and South of Brazil).

In relation to the sex estimation of the Toca dos Coqueiros individual, we will assess it based on craniometric comparisons with other early and late Holocene individuals from CESA. Based on pelvic features (presence of a ventral arc, and wide and deep preauricular sulcus) and DNA analysis, the individual from Toca dos Coqueiros was first described as a female^52^. This was done despite the authors’ recognition of the bad preservation of some features (e.g., the greater sciatic notch), as well as their description of other features as presenting a probable male morphology (e.g., the thickness of the supraorbital margin). Moreover, since there are no detailed descriptions of the sex determination that were done through the DNA analysis, and the preservation of collagen for this is very unlikely, this result has been repeatedly questioned^27,53^. Based on the study of pelvis, cranium, mandible, and long bone features, as well as assessments based on biological distances, “Zuzu” has been defined by some authors as a gracile male^27,53,54^, although some studies have also considered the individual as undetermined^51^. However, so far, the sex of this individual has not been assessed in comparison to other individuals from the region in a systematic way. This approach is needed since South American early Holocene individuals have been described as very gracile, especially those coming from the Central-East region^27,55,56^. This might have an impact on the pattern of sexual dimorphism in such a way that it differs from the one characterizing late Holocene groups, i.e., craniofacial variation is associated with strong sex differences due to the larger size and more robust features of males^57^. By comparing the Toca dos Coqueiros cranial variation with that of early and late Holocene individuals from Central-East of South America, we intend to provide a more accurate sex estimation appraisal based on geographical and chronological criteria.

Finally, we will evaluate the morphological similarities and differences of the individual from Toca dos Coqueiros with other samples from the late and early Holocene of Brazil. Previous morphometric studies of the Toca dos Coqueiros individual showed that it presents craniometric affinities with Austro-Melanesians, as well as other early Holocene individuals from Lagoa Santa, Capelinha (Brazil), Sabana de Bogotá (Colombia), Mexico Basin (Mexico), and Palli Aike (Chile)^27,58,59^. However, until now, there have been no studies evaluating the craniometric variation of Serra da Capivara individuals with late Holocene groups from the region, i.e., CESA (but see^60^, in which the early Holocene is represented by individuals from Lagoa Santa). As recent studies show, taking a regional perspective is relevant for addressing biological affinities^10,14,19,21^, which otherwise could be obscured when applying a continental or worldwide approach. Additionally, studying craniometric variation within a region becomes relevant since the skull provides information on some of the multiple factors that shaped phenotypic variation in South America (e.g., migration, selection, drift)^7,10,13,18–23^. In addition, we will address the temporal and spatial patterns of morphological differences and similarities in CESA since we consider this regional perspective as a necessary step to better understand the diversification of *Homo sapiens* in the continent. Getting a deeper understanding of the cranial variation in samples from Central East South America contributes to discussing the number of ancestral lineages from which they originated, as well as the magnitude of variation of the founding population.

## Results

### Chronological assessment: relation to other early Holocene radiocarbon-dated skeletons

**Figure 2:**
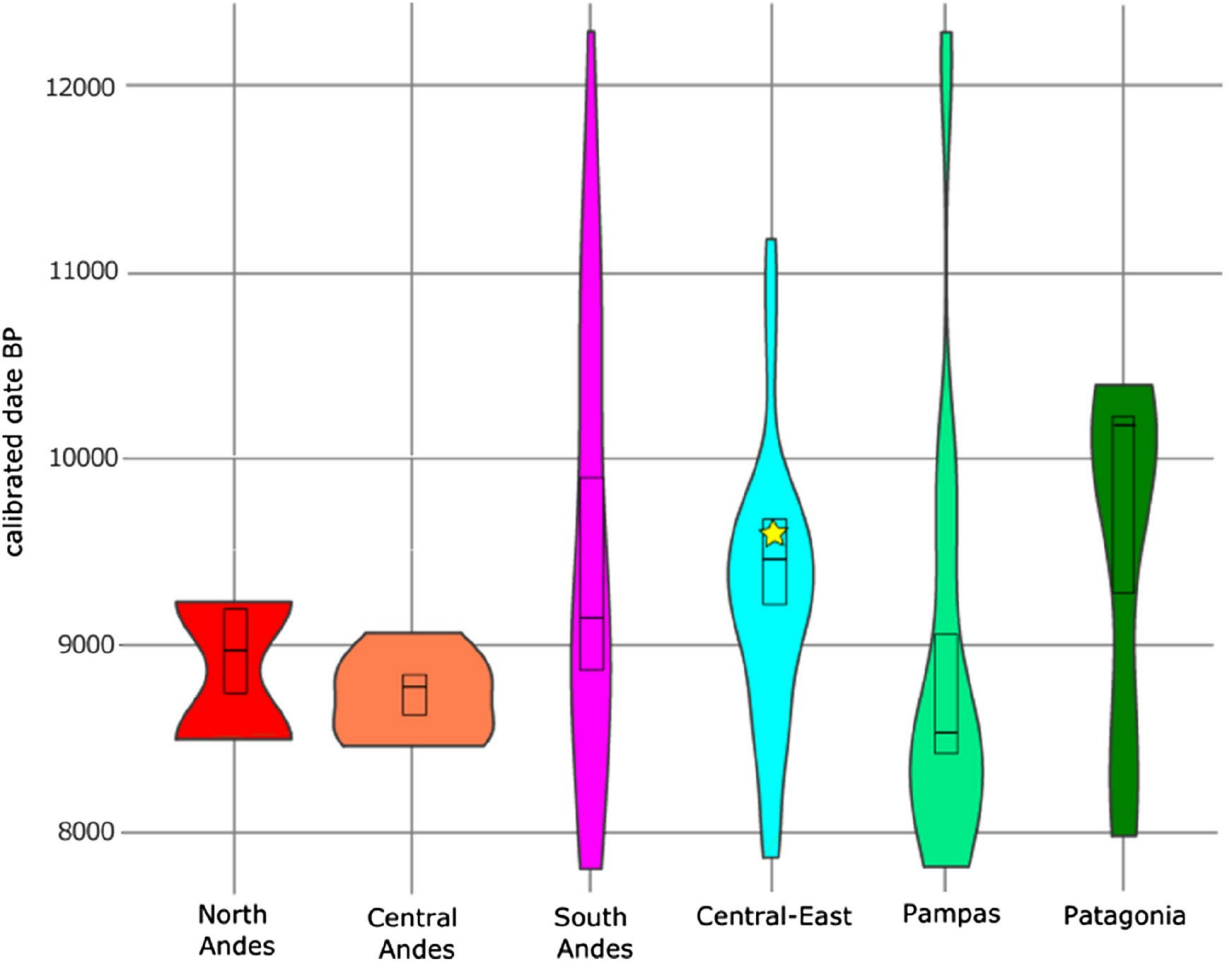
Violin plots showing the distribution of direct radiocarbon dates on early Holocene human bone/teeth per region in South America, based on the 2-sigma error of the calibrated date range. The yellow star indicates the radiocarbon date of Toca dos Coqueiros skeleton. The boxplot within each violin shape indicates the mean and standard deviation for that group.

The radiocarbon date we obtained from the dental enamel carbonate of the Toca dos Coqueiros individual (Lab code: BETA–536529; 8,640 ± 30 ^14^C years BP; 2-sigma 9,526–9,681 cal years BP; mean: 9,603 cal years BP) falls within the range of those considered as reliable early Holocene radiocarbon dates (i.e., older than 7000 ^14^C years BP) obtained directly from human skeletons recovered at archaeological sites in South America (Fig. 2; Table S1). This result should not be taken as definitive but rather as a minimum age that could be older if chemical alterations induced by the surrounding environment from the burial can be disregarded. Considering that under temperate/wet conditions, dental enamel carbonate is not exempt from isotopic exchange^61^, the original carbonate might rather be a mix between biogenic carbonate and secondary carbonate derived from the CO_2_ of the local atmosphere during combustion. This gives the altered bone samples younger ages than their actual ages (limited to 300 ^14^C years or less for the Holocene^61^). Even though the dental enamel from the individual of Toca dos Coqueiros looked dense, white, without any discoloration, and that pretreatment methods were applied to remove possible intrusions (see “Methods”), we cannot discard the possibility of alterations to the carbon, and therefore cannot reject the possibility that the chronology associated with this individual might be a slightly older than what is reported here^62^.

The violin plot shows a normal distribution of the radiocarbon dates from CESA (N = 44). This means that most of the available radiocarbon dates fall within a small range of values with one peak and few outliers. The date we report here (9,603 cal years BP), falls slightly above the mean (∼9,450 cal years BP), but also very close to it, as well as to the data peak for radiocarbon values in the region (Fig. 2). Possible outliers in the region can be identified with radiocarbon dates older than 10,500 cal years BP. The regions of the Central Andes (N = 6) and North Andes (N = 2) show a uniform and bimodal distribution respectively, which could be interpreted as a result of their small sample size. The other regions have larger sample sizes (Pampas, N = 23; Patagonia, N = 12; South Andes, N = 30) and present more elongated distributions without a peak, depicting a larger range of variation in which the dates are equally distributed. Given that there is only one date available for the Lowlands (Table S1; 7,817 cal years BP), it was not possible to include this region in the violin plot, since at least two values are needed for such plots. The radiocarbon date mean from CESA is the second oldest after Patagonia and presents the largest number of direct radiocarbon dates for South American late Pleistocene/early Holocene sites. The Pampas and Southern Andes regions present the earliest dates on the continent, although for the latter, those early dates are isolated, i.e., there are just a few radiocarbon samples directly dated, indicating either that more research is needed in those areas or the occupation density there was lower. Similarly, the only direct radiocarbon date for the Lowlands may result from the poor preservation conditions of collagen in the tropics.

### Sex estimation based on craniometrics analysis of Toca dos Coqueiros

To assess the biological sex of the individual from Toca dos Coqueiros, we conducted two Discriminant Function Analyses. In the first analysis, discriminant functions were calculated for the individual from Toca dos Coqueiros in comparison with both, early and late Holocene individuals of the comparative sample (Table 1; Guajajara, Sambaqui Santa Catarina, Tupi-Guarani, Sambaqui Rio de Janeiro, Botocudo, Lagoa Santa). The results showed an overall correct classification rate for sex of 71.3% and 62.96% when cross-validated, and that the individual from Toca dos Coqueiros was classified as a female (Table 2). Of the whole dataset, 75% of the female individuals, and 70.5% of the male individuals were correctly classified (Table 2). In the second analysis, discriminant functions were calculated for the individual from Toca dos Coqueiros in comparison with the early Holocene individuals from Lagoa Santa exclusively. According to the results of this analysis, the individual from Toca dos Coqueiros was classified as a male. In this case 83.3% of the female individuals, and 74.1% of the male individuals were correctly classified, whereas the overall correct classification rate was 87.18% and 76.92% when it was cross validated (Table 2). Due to the highest correct classification rates of the last analysis, and the fact that sexual dimorphism patterns might differ among early Holocene individuals, we consider the sex estimation of the Toca dos Coqueiros individual as a male as the most accurate one.

**Table 1:** Comparative samples from Central-East South America used for the morphometric analysis.

Series	Area in Central-East South America	Number of individuals	Sex proportion per sample (M/F)	Chronology
Tupi Guarani (Pará State)	North	5	5/0	Late Holocene
Toca dos Coqueiros	North-East	1	?	Early Holocene
Guajajara	North-East	12	12/0	Late Holocene
Lagoa Santa	Central-East	39	27/12	Early Holocene
Botocudo	Central-East	18	10/8	Late Holocene
Sambaqui (Rio de Janeiro)	South-East	12	12/0	Late Holocene
Sambaqui (Santa Catarina)	South	22	22/0	Late Holocene
**Total**	**–**	109	89/20

**Table 2:** Results of the discriminant function analysis.

	First analysis			Second analysis		
	M	F	Total	M	F	Total
M	62	26	**88**	20	7	**27**
F	5	15	**20**	2	10	**12**
Total	67	41	**108**	22	17	**39**
Correct classification rate	**71.3% (62.96%)**			**87.18% (76.92%)**		

The first analysis was performed using all comparative samples, the second one using only the Lagoa Santa series as a comparative sample (see main text). The percentages in parenthesis correspond to the cross-validated rate of correct classification.

### Morphological variation of the Toca dos Coqueiros’ skeleton in relation to other samples from Brazil

**Figure 3:**
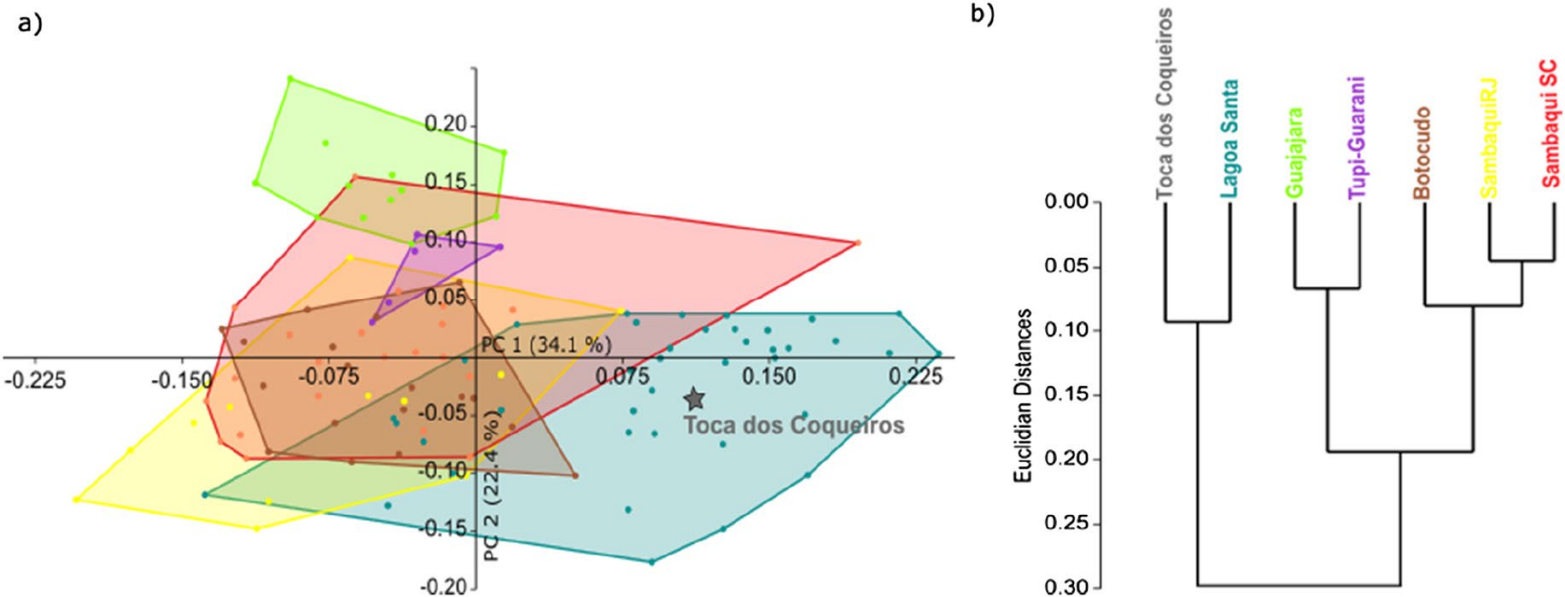
Morphometric results: (**a**) PCA showing the distribution of samples along the PC1 (Principal Component 1) and PC2 (Principal Component 2) of the cranial shape variables. Convex hulls indicate populations. Late Holocene samples: Guajajara (green), Sambaqui SC (red), Sambaqui RJ (yellow), Tupi-Guarani (purple), Botocudo (brown); early Holocene samples: Lagoa Santa (turquoise). The individual from Toca dos Coqueiros is highlighted with a star symbol; (**b**) Cladogram showing the results of the Ward’s hierarchical clustering analysis.

We conducted a Principal Components Analysis (PCA) to explore the cranial shape variation of the individual from Toca dos Coqueiros in relation to other early and late Holocene individuals from CESA. The plot of the first two PCs, which together explain 56.5% of variance, shows that the morphological variation of the samples analyzed is firstly structured according to differences in chronology, and secondarily by geographical provenance (Fig. 3). The separation between the early and the late Holocene populations is clearer along the second PC, but also partially recognizable along the first one, on which the earliest individuals fall mostly within the bottom right corner of the plot, whereas the more recent populations overlap on the top left corner. We can observe that the individual from Toca dos Coqueiros groups with the Lagoa Santa series along the first two PCs, falling in the center of the Lagoa Santa sample’s distribution (Fig. 3). Some of the Botocudo individuals overlap with the distribution of early Holocene ones (i.e., Lagoa Santa, Toca dos Coqueiros), while others are more similar to the late Holocene ones. The Guajajara sample, which is the northernmost region in our sample, is located on the positive extreme of PC2, while the southernmost sample we include, Sambaqui Santa Catarina, is the closest to the negative extreme (not including the early Holocene individuals). The PC loadings show that the variance explained by these PCs corresponds mainly to the length and height of the vault, as well as to the facial width (Table 3). This points to a craniofacial morphology among Toca dos Coqueiros and the Lagoa Santa individuals that is characterized by antero-posteriorly elongated and narrow vaults, as well as low and wide faces, whereas the morphology of the late Holocene populations, particularly the Guajajara individuals, is characterized by shorter and wider crania, and higher and narrower faces.

**Table 3:** Principal component loadings.

	PC 1 (34.1%)	PC 2 (22.4%)	PC 3 (16.1%)	PC 4 (11.6%)	PC 5 (6.3%)	PC 6 (3.5%)	PC 7 (2.7%)	PC 8 (1.8%)	PC 9 (1.1%)	PC 10 (2.3 E-16%)
NL	0.6036	0.2832	− 0.5385	0.2863	− 0.1068	− 0.2520	− 0.0200	− 0.0774	0.0474	0.3162
NW	− 0.1735	0.5004	− 0.0342	− 0.7362	− 0.0331	− 0.1206	− 0.0373	− 0.2386	0.0481	0.3162
FW	− 0.5419	− 0.4626	− 0.6137	0.0037	0.0231	− 0.1084	0.0440	− 0.0372	− 0.0064	0.3162
MNH	0.3909	− 0.6056	0.3029	− 0.3244	− 0.3451	− 0.1974	0.1136	− 0.0262	0.1086	0.3162
FH	− 0.3580	0.1841	0.4025	0.4199	− 0.2366	− 0.5173	− 0.2455	0.0664	0.1069	0.3162
RH	− 0.0894	0.1052	0.1959	0.2792	0.0106	0.2646	0.7049	− 0.4413	− 0.0537	0.3162
RW	0.0180	0.0873	0.0437	− 0.0514	− 0.0622	0.0984	0.0943	0.5316	− 0.7632	0.3162
OH	− 0.0161	0.1087	0.0063	− 0.0086	0.0962	0.3362	0.1391	0.6041	0.6175	0.3162
OW	0.0178	− 0.0554	0.0452	0.1163	− 0.2139	0.6276	− 0.6009	− 0.2791	− 0.0504	0.3162
AW	0.1485	− 0.1452	0.1901	0.0151	0.8677	− 0.1310	− 0.1923	− 0.1023	− 0.0548	0.3162

*NL* Neurocranial length, *NW* Neurocranial width, *FW* Facial width, *MNH* Midneural height, *FH* Facial height, *RH* Respiratory height, *RW* Respiratory width, *OH* Optic height, *OW* Optic width, *AW* Alveolar width.

In addition, we assessed the magnitude of phenotypic distances among samples by calculating Euclidean Distances, which were used to perform a Ward's hierarchical clustering analysis. The resulting cladogram shows chronological and geographical grouping of the samples. The individuals from Toca dos Coqueiros and Lagoa Santa, from the early Holocene, cluster independently from the late Holocene groups stemming from a separated node (Fig. 3b). The late Holocene groups are divided into two clusters, one containing samples from the South and Central-East of Brazil (Botocudo, Sambaqui Rio de Janeiro, Sambaqui Santa Catarina) and a cluster including samples from the Northeast (Guajajara, and Tupi Guarani from the state of Pará) of Brazil. The Euclidian distances confirm these trends, showing that the cranial morphology of the individual from Toca dos Coqueiros is more similar to that of the Lagoa Santa individuals, while it substantially differs from that of the Guajajara population, despite the fact that the latter comes from the same region, i.e., northeast of Brazil (Table 4). In fact, the Toca dos Coqueiros and the Guajajara samples are the ones that differ most of all the samples studied, while the most similar are the samples from Sambaqui Rio de Janeiro and Sambaqui Santa Catarina, which are geographically very close to each other (Table 4).

**Table 4:** Euclidean distances among samples.

	Toca dos Coqueiros	Lagoa Santa	Sambaqui (Rio de Janeiro)	Guajajaras	Sambaqui (Santa Catarina)	Tupi Guarani	Botocudo
Toca dos Coqueiros	0	0.1313	0.2442	0.2832	0.2177	0.2143	0.2187
Lagoa Santa	0.1313	0	0.1868	0.2311	0.1568	0.1658	0.1644
Sambaqui (Rio de Janeiro)	0.2442	0.1868	0	0.2131	0.0639	0.1566	0.0948
Guajajaras	0.2832	0.2311	0.2131	0	0.1556	0.0941	0.1901
Sambaqui (Santa Catarina)	0.2177	0.1568	0.0639	0.1556	0	0.1035	0.0795
Tupi Guarani	0.2143	0.1658	0.1566	0.0941	0.1035	0	0.1372
Botocudo	0.2187	0.1644	0.0948	0.1901	0.0795	0.1372	0

## Discussion

We present radiocarbon and morphometric analyses of the first early Holocene human skeleton from Serra da Capivara, Northeast of Brazil, to be directly dated. This is one of the few archaeological localities in South America that presents a high number of human burials associated with late Pleistocene/early Holocene evidence^39^. After 50 years of unprosperous attempts, we were able to select samples of dental enamel that have been successfully directly radiocarbon dated. We obtained a radiocarbon date (9,603 cal years BP) that falls within the expected range of values for the region, presenting a slightly older (but very close) value to the mean of CESA. Despite this date falling within the range of reliable ones for South America, it should be taken as a minimum age, due to the potential isotopic exchange during fossilization that can produce overly young dates, as has been previously reported^61,62^. In addition, the calibrated mean age from the Toca dos Coqueiros individual (mean: 9,603 cal years BP; 2-sigma 9,526–9,681 cal years BP) is ∼1,400 years younger than the dating that was previously obtained from the charcoal associated with this skeleton (mean: 11,022 cal years BP; 2-sigma 11,070–10,975 cal years BP). These are the only two dates available so far, both supporting Zuzu’s early Holocene chronology. As we showed here, CESA is the only area in the continent presenting a normal distribution of radiocarbon dates performed directly on human skeletons. This means that most of the radiocarbon dates that are available for this area cluster close to the central peak of ∼9,450 calibrated years BP. The reasons behind this chronological range occurring more frequently are currently unknown. We could hypothesize that this may represent a chronological range characterized by high population density in comparison to immediately previous and later times, but it could also be the result of better archaeological visibility, differential preservation conditions, and/or methodological biases in the research design, as has been suggested before^34,63^. The radiocarbon date we obtained for the Toca dos Coqueiros individual is slightly older than the central peak for the region, with a 2-sigma range of 9,526–9,681 calibrated years BP. We are aware that obtaining a series of radiocarbon dates for this skeleton would make our results more robust (sensu^39^), however, unfortunately this has not been possible to date. Considering that it took several decades to find a suitable method for dating the skeletons coming from this region, in which collagen is not preserved in the bones, we think of this achievement as a milestone that should be considered in future debates on the first human expansions in South America, as well as on the biological variation of the first inhabitants in the continent. Future studies in the archaeological sites at Serra da Capivara should focus on applying the enamel dental carbonate and any other method of direct radiocarbon dating possible, allowing the comparison of those possible new dates with the dental enamel carbonate dating we report here.

With regard to the intense debate about the sex determination of the Toca dos Coqueiros individual^27,52–54^, unfortunately, our results are not conclusive on this matter. The discriminant functions that we calculated showed that when the craniometric variation of the Toca dos Coqueiros individual was compared to that from the other early Holocene individuals, it classified as a male, but when it was compared to all the samples from CESA together (early and late Holocene individuals), it was classified as a female. It should be noted that our first analysis was carried out with a small number of females, and this could bias the results. However, since the classification rate was higher for the former, we rely more on this result, considering the Toca dos Coqueiros individual as a probable male. This result is also supported by the presence of goods associated to this burial, i.e., bifacial projectile points, which are tools used for hunting, an activity that is usually linked to male sex individuals^64^. However, it is worth noting that some recent studies argue that hunting was a gender-neutral activity during the early Holocene in South America^65^. Despite the male estimation that we obtained contradicting the previous genetic results for this individual, which might have potential problems since the preservation of collagen is very unlikely^52^, it is indeed supported by previous morphometric analyses and morphological descriptions, which were based not only on cranial features, but also on sexually dimorphic features of the pelvis^27,53,54^. This is relevant since the pelvis is considered the most sexually dimorphic structure in the skeleton, and therefore, the most reliable in terms of sex determination^66^. Considering that the individuals from the early Holocene of South America have been previously described as being more gracile than the late Holocene ones, this might explain the disagreement regarding the sex determination of this individual when compared to other early and late Holocene ones^27,53^. For instance, another presumably early Holocene skeleton from Serra da Capivara, coming from the site Toca da Janela da Barra do Antonião, and known as "Zazá", has been described as one of the most gracile individuals, presenting smaller bone dimensions than Zuzu^56^. The morphological differences between these two skeletons may either demonstrate the pattern of sexual dimorphism in the early Holocene (“Zazá" being a female and “Zuzu” a male) or it could result from the idiosyncratic variation among individuals of the same biological sex (both being female). Unfortunately, the degree of conservation of dimorphic sexual characters is reduced in early Holocene samples as it depends on the general preservation of bones. Further studies are needed to address this issue, probably by comparing the morphological variation of sexual dimorphism in the pelvis and skull of “Zuzu” in relation to other individuals from the early Holocene.

Our results show that the individual from Toca dos Coqueiros presents greatest morphological similarities with the individuals from Lagoa Santa, which is the other early Holocene archaeological locality in our comparative sample from CESA. This is illustrated by the PCA, the Ward’s hierarchical clustering results, and the Euclidean distance matrix (Fig. 3a,b, Table 4). This result, as well as other morphometric results of the present study, should not be taken as definitive but as limited to the research design of our study which includes only early and late Holocene individuals from CESA. The magnitude of morphological differentiation that exists among early Holocene individuals, as well as among late Holocene ones, provides clues of the diversification process undergone by the earliest inhabitants of the continent^5,22,67–70^. This study shows that some late Holocene samples from geographically close regions present stronger morphological similarities to each other (Sambaqui Rio de Janeiro, Sambaqui Santa Catarina), while other late Holocene samples from distanced geographical localities in CESA are morphologically highly different from each other (Sambaqui Rio de Janeiro, Guajajara). The largest morphological differences among late Holocene populations could be the result of independent lineages diversifying across time, isolated geographically and reproductively from each other, and adapting to different environments^71^. On the contrary, the morphological similarities existent among the individuals coming from the Sambaquis could be explained by a more recent common ancestor or frequent gene flow among them^72^. The biological distance between the early Holocene individuals (e.g., the individual from Toca dos Coqueiros and the sample from Lagoa Santa) falls in an intermediate position between the more disparate late Holocene groups and the very similar ones, meaning that the morphological variation of the earliest individuals from CESA is moderate in this context. This could be interpreted as resulting from relatively high morphological variation in the ancestral population from which they diverged, as is supported by previous studies^5,22,67,68,73–75^.

The individual from Toca dos Coqueiros presents the strongest morphological differences from the Guajajara individuals, despite both samples coming from the same area, i.e., Brazilian North-East. This result does not agree with the presence of biological continuity of prehistoric human populations in CESA, but rather suggests a contribution of other lineages into the recent (i. e., late Holocene) configuration of populations in the area. Similarly, this study shows that there is general morphological differentiation between early and late Holocene individuals across Brazil. This is supported by the results of our clustering analysis, in which the early Holocene individuals from Lagoa Santa and Toca dos Coqueiros form a separate cluster differing from all the late Holocene samples. However, these interpretations are limited to the results obtained in this study and should be further tested with a larger sample in future projects. The individuals from Toca dos Coqueiros and Lagoa Santa present a craniofacial morphology characterized by antero-posteriorly elongated and laterally narrow cranial vaults, as well as low and wide facial skeletons. This phenotype has already been described multiple times for other early Holocene individuals from South America^2,5,11,15,18,19,21,26,40,58,60^, and in general differs from the cranial morphology that is present in most of the late Holocene individuals, although there are a few exceptions to this trend (see^13,30,76,77^). This distinctive morphology might represent the generalized phenotype characterizing the first populations arriving on the continent, or at least the phenotype that is present in the early Holocene individuals from CESA. It still remains uncertain which are the evolutionary processes that shaped the morphological differences that we found between early and late Holocene individuals. Answering this issue goes beyond the scope of the present study, but as currently understood, either the late Holocene populations diverged directly from the earliest ones evolving locally^7,14,77,78^, or the recent groups derived from a more recent population lineage arriving later to the region^15,25,79^. Since recent studies show strong affinities between early and late Holocene individuals, which might imply probable biological continuity between them, the discussion of their common ancestor is currently centered on disentangling if the ancestral population from which both diverged comes from east Asia, Beringia, or from North America^80–83^. Elucidating if there was more than one ancestral population that gave rise to native Americans requires further work, not only incorporating more directly dated human remains into the comparisons, but also making interdisciplinary efforts to tackle this research problem. Future studies should focus on testing this hypothesis by comparing samples from all these regions together in a comprehensive study.

## Conclusion

In this paper we showed that the first radiocarbon-dated individual from Serra da Capivara, Brazil, shows strongest craniometric affinities with other early Holocene individuals from CESA, i.e., Lagoa Santa, and strongest differences from late Holocene individuals from the same region, i.e., Guajajara. Additionally, we found that the morphological variation among individuals from the early Holocene of CESA is moderate when compared to the variation present among late Holocene groups. Thus, this study supports the hypothesis that the morphological variation of the earliest inhabitants of South America was moderate and differs from the level present in the most recent individuals, suggesting either the arrival of more than one lineage and/or an initial heterogeneous founding population. We also showed that the direct radiocarbon dating that we obtained falls within the expected ages for early Holocene individuals from South America, and that the sex estimation of this individual differs depending on whether it is compared to early or late Holocene individuals. We expect that our work will contribute to the current debate on the human diversification in the Americas by adding chronological and morphometric results from an individual and geographic area (Serra da Capivara), that have been previously dismissed or excluded from international debate. Incorporating these results into the current discussion will allow some of the current difficulties to be overcome, by expanding a sample that has over-relied on the study of the same few individuals, repeatedly analyzed with the complete methodological toolkit available to date. Future research should focus on studying in detail further individuals from Serra da Capivara in a comparative context of individuals from Brazil, South America, and other continents as well. Interdisciplinary efforts in which multiple strands of evidence is carefully combined are needed to move forward into a more comprehensive debate on the first humans’ expansions into South America.

## Methods

### Sample: “Zuzu”, the individual from Burial 1 of Toca dos Coqueiros, Serra da Capivara

The National Park Serra da Capivara, located in the southeast of Piauí state, northeastern Brazil (Fig. 1), has more than 1300 reported archaeological and paleontological sites. The research in this area started in the 1970s, it was led by one of the authors (NG) and supported by the Franco-Brazilian Mission and the Fundação do Museu do Homem Americano (FUMDHAM). As a result of systematic prospection and excavations, the known paleontological and archaeological records are extremely rich, and the area was first recognized as a Brazilian National Park and later as an UNESCO heritage site^84^. From the archaeological sites reported, approximately 27 include human burials, of which, prior to the current analyses, seven present indirect but associated radiocarbon dates representing late Pleistocene/early Holocene human occupations: Toca do Garrincho (acid washes from teeth, 12,170 ± 40 BP;^85^), Toca da Janela da Barra de Antonião (charcoal, 9,670 ± 140 BP;^56^), Toca do Paraguaio (sediment, 8,670 ± 120 BP;^86^), Toca da Cerca do Elias (charcoal, 10,270 ± 35 BP;^85^), and Toca dos Coqueiros (charcoal, 9,870 ± 50 BP;^64^). Unfortunately, due to the lack of collagen preservation, there have been no direct radiocarbon dates available until now^39^.

**Figure 4:**
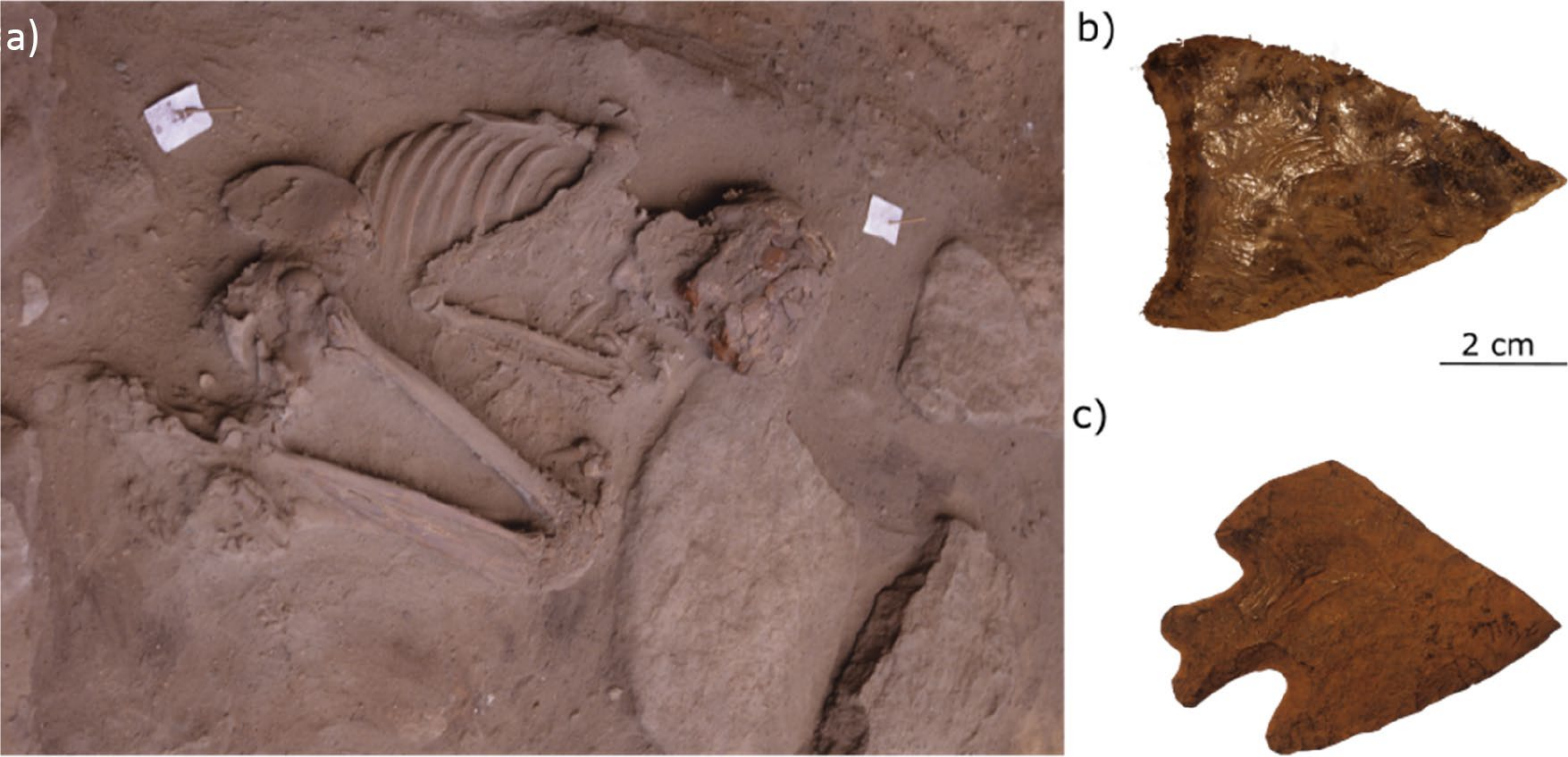
Burial 1 from Toca dos Coqueiros, Serra do Capivara, Brazil: (**a**) Skeleton position; (**b**) Bifacial projectile point made of hyaline quartz; (**c**) Stemmed projectile point made of chert. This Figure has been created from images that belong to the FUMDHAM archives.

The archaeological site of Toca dos Coqueiros, located in the Serra da Capivara National Park (8° 50.290’ South, 42° 33.739’ West), was excavated between 1995 and 1997 by a team of archaeologists from the FUMDHAM^63,85,87^. It consists of a single burial situated in a small rock shelter with rock paintings and marked by large and middle-sized rocks located on top and the sides^64^ (Fig. 4a). The skeleton, which was found fully articulated and almost complete, was placed in an oval fossa lying on its left side in a tightly flexed position^52^ (Fig. 4a). It was accompanied by grave goods that are not very frequent in the area: two bifacial projectile points, as well as 15 flakes, and four plano-convex scrapers^64^ (Fig. 4b,c). Also, bones of small animals, vegetal remains, and a human hair not associated with the skeleton, were found nearby. The skeleton was firstly excavated on-site, then exhumed together with the block of sediment surrounding the burial, and after being consolidated with acryloid B-72, it was wrapped to finish the excavation in the laboratory^64^.

**Figure 5:**
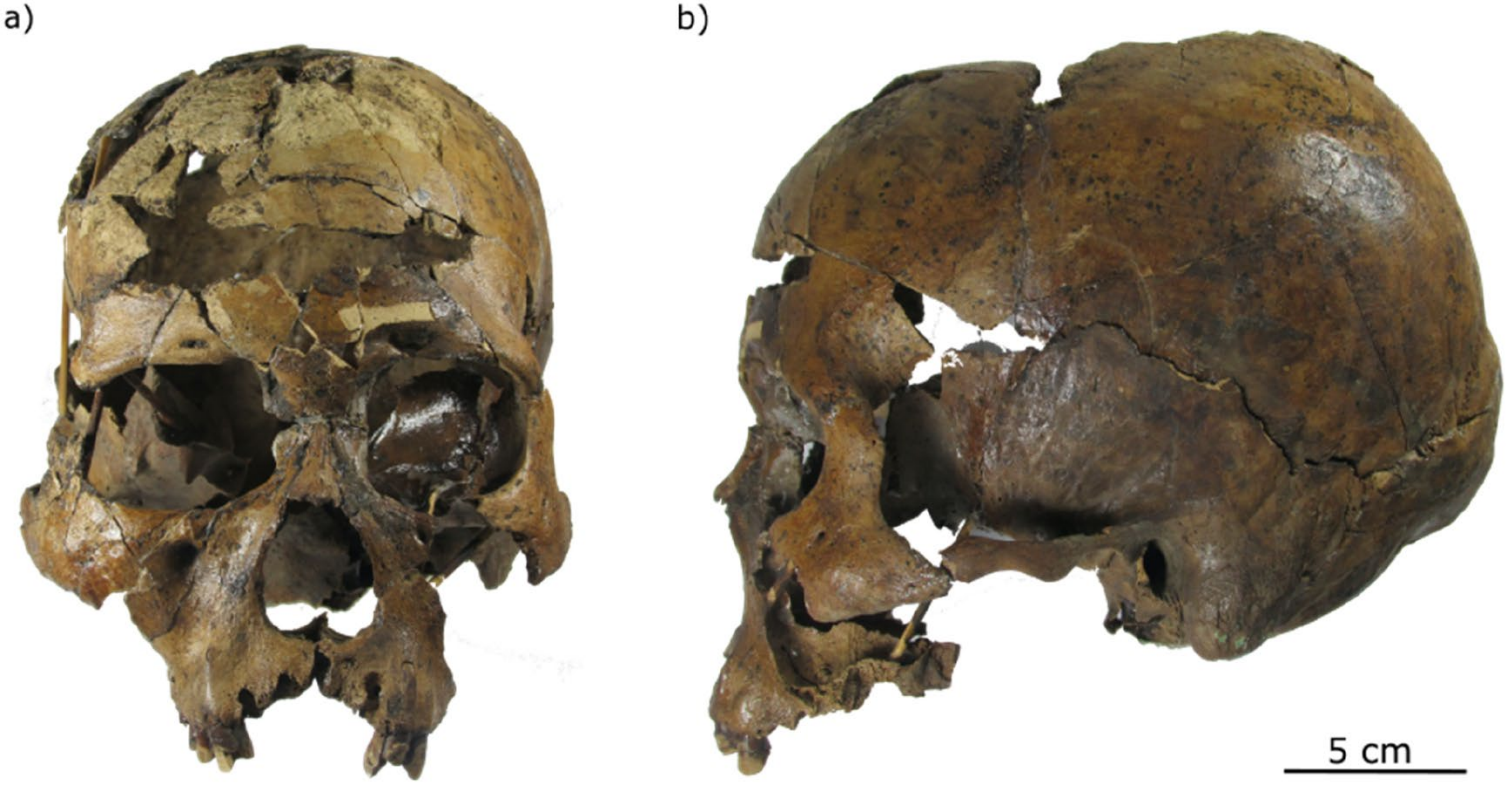
The Toca dos Coqueiros skull: (**a**) frontal view; (**b**) lateral view. This Figure has been adapted from images that belong to the FUMDHAM archives.

The skull, which was reconstructed and curated by Nelson (2005), was described as presenting no evidence of artificial or taphonomic deformation (Fig. 5;^27,53^). Previous osteobiographic studies have estimated age and stature, recorded the presence of paleopathology, and determined the sex^27,52–54,88^. Overall, these studies have agreed that the individual is a middle adult (35–45 years), although the sex determination is still under debate (see “Introduction”).

Several attempts were made to extract collagen from the bones of the Toca dos Coqueiros’ skeleton for radiocarbon dating, but since they all failed until now, we tested the availability of datable material in the tooth enamel carbonate. The only dates available prior to the current dental enamel carbonate analyses are indirect and correspond to charcoal associated with the calcaneus (BETA-109844: 9,870 ± 50 BP; 11,120–11,025 cal years BP;^64^), and a hair infected with louse eggs (BETA-104571: 10,640 ± 80 BP; 12,743–12,465 cal years BP^64,89^), although the later may not belong to this individual^52^. Even though these dates are indirect, and therefore not considered appropriate for the inclusion of this skeleton among the ancient Paleoindian contexts of Brazil^39^, its cranial morphology shows similarities with other early Holocene human groups that populated the American continent^27,52–54,58,59^.

### Radiocarbon analysis and comparison with other direct radiocarbon dates

A tooth crown (left M^3^) and teeth fragments (left M^1^, mandibular molar) were selected to perform radiocarbon analyses from the dental enamel carbonate. The teeth were grouped into one sample (BETA—536529) that was analyzed in the laboratory Beta Analytic Inc. (Florida, USA). Once in the specialized lab, the dental enamel was abraded to remove any attached surface particles or adhesions. It was then pretreated with 1.2 N HCl to clean the external surfaces and remove any probable secondary carbonate, rinsed to neutral with deionized H_2_O, and vacuum desiccated until dry. Finally, it was crushed to a powder and acidified under vacuum with 80% phosphoric acid in order to collect the evolved CO_2_ for the subsequent analysis by Isotope-ratio mass spectrometry (d13C IRMS), while it was graphitized for the subsequent accelerator mass spectrometry (AMS) detection.

We present here the conventional radiocarbon age, as well as the 2-sigma error after conversion to calendar years using the database SHCAL13 with the online software Calib Rev 8.2^90^. We constructed a violin plot to evaluate how the Toca dos Coqueiros radiocarbon date fits within the distribution of direct radiocarbon dates of early Holocene skeletons across different regions in South America (Fig. 1; Table S1). A violin plot, which is a combination of a box plot and a Kernel density plot, shows the distribution of quantitative data across several groups by featuring a density estimation of the underlying distribution. Comparative data comprise the 2-sigma error of the calibrated radiocarbon dates from other direct radiocarbon dates reported for skeletons from South America, which were obtained from the literature^24,37^. The full list of 119 direct radiocarbon dates can be found as Supplementary Table S1 online. We constructed the violin plot using the ggplot2 package in R 4.1.0^91^.

### Morphometric analysis

**Figure 6:**
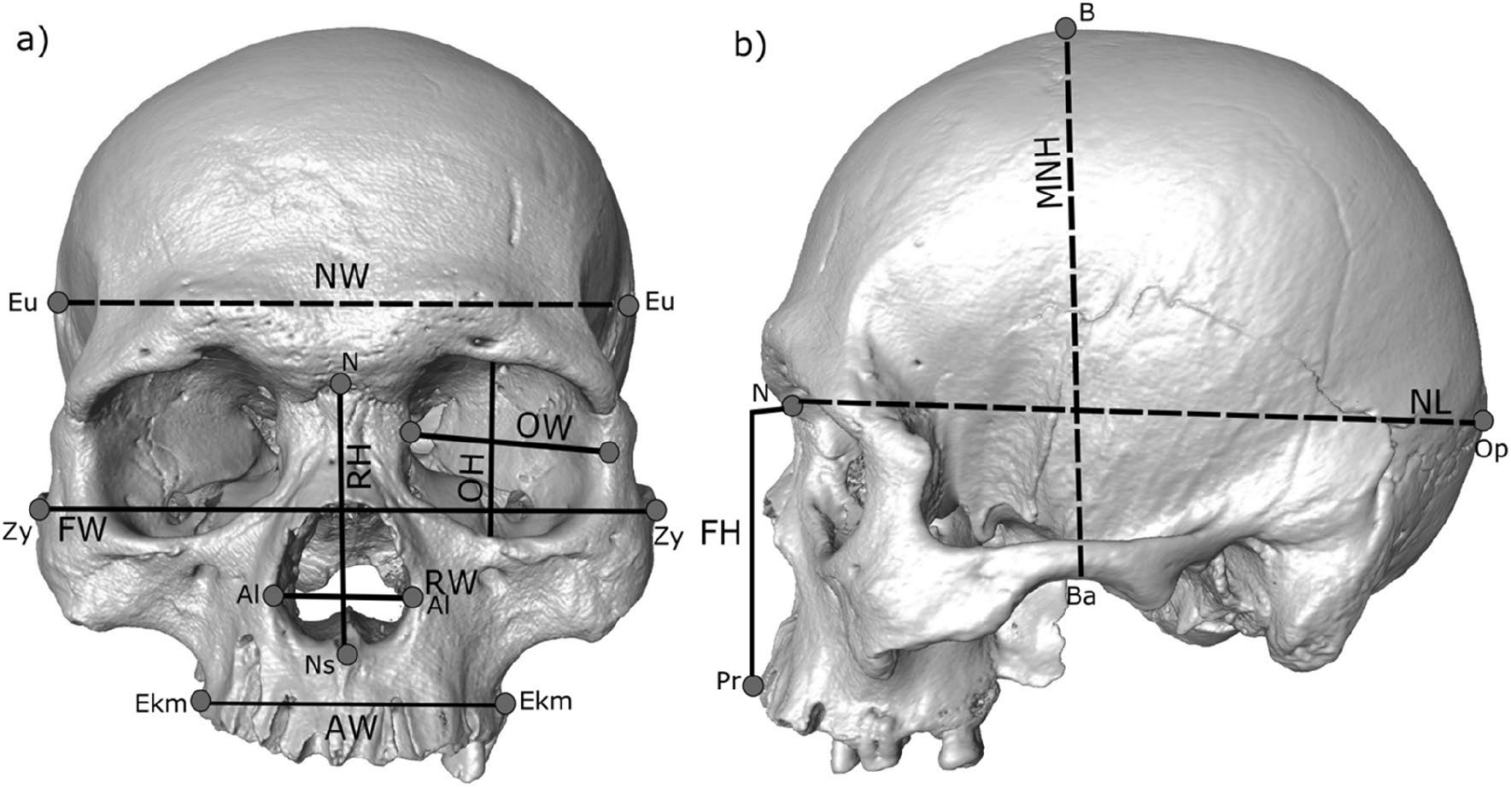
Craniofacial measurements used for this study (see Table 3) in frontal (**a**) and lateral view (**b**). Solid lines indicate facial skeleton measurements, and dashed lines, neurocranial measurements. References: NW = Neurocranial length (Eu: Eurion—Eu: Eurion); FW = Facial width (Zy: Zygion—Zy: Zygion); AW = Alveolar width (Ekm: Ectomolare—Ekm: Ectomolare); RW = Respiratory width (Al: Alare-Al: Alare); RH = Respiratory height (N: Nasion—Ns: Subespinale); OW = Optic width (D: Dacryon—Ec: Ectoconquion); OH = Optic height (maximum height from the upper to the lower orbital borders perpendicular to the horizontal axis of the orbit); NL = Neurocranial length (N: Nasion—Op: Opistochranion); MNH = Midneural height (Ba: Basion—B: Brema); FH = Facial height (N: Nasion—Pr: Prosthion). Figure generated with Inkscape v.1.1.2 (https://inkscape.org/release/inkscape-1.1.2/).

A total of ten linear measurements were used to describe cranial shape variation within the sample (Fig. 6). Some of the measurements describe the whole skull, while others represent local areas such as the orbits and the nose (Fig. 6). Measurements on the skull from Toca dos Coqueiros were taken by one of the authors (SFSMDS) following the recommendations by Buikstra and Ubelaker^92^, by using analogic (Mitutoyo, 150 mm) and digital calipers (Mitutoyo No. 500-144, 150 mm). The permission for studying this sample has been requested to FUMDHAM and approved prior to its study.

For establishing morphometric comparisons between the skull from Toca dos Coqueiros and other early and late Holocene individuals, we used Pucciarelli’s database^23,93^. This is the largest freely-available database including cranial measurements from thousands of individuals from South America. For this study, we selected 108 early and late Holocene adult individuals from six populations of the Central-East of South America, the area where Toca dos Coqueiros is located (Table 1). Most individuals measured by Pucciarelli are males, except for the series that are significant in terms of early morphological variation, which consist of both males and females. This is the case for the Lagoa Santa and Botocudo series, which here are considered relevant due to their characteristic “Paleoamerican morphology” (i.e., presence of cranial features such as long cranial vault and narrow facial skeleton, among others). In both cases sex was determined by examining dental maturation and fusion of cranial sutures, as well as the macroscopic criteria of sexually dimorphic cranial features suggested by Buikstra and Ubelaker^92^. Considering that the sex of Toca dos Coqueiros is still under discussion, by including both females and males in our analysis we expect to cover a large spectrum of morphological variation for the earlier individuals of the area.

Since we combined morphometric measurements taken by two observers (Héctor Pucciarelli and SFSMDS), we tested for the existence of significant differences to detect if the results may differ due to interobserver error. For this, we compared the differences between the two observers for the 10 measurements used in this study taken on a series of 5 individuals from Museo Nacional of Brazil. Repeated measures ANOVA and the Intraclass Coefficient showed no significant statistical differences between the sets of measurements that were registered by the two observers (F = 0.28, ICC = 0.99, p = 0.61).

The raw measurement variables from the complete sample were standardized by using the arithmetic mean to obtain Mosimann ratios, following previously described procedures^94,95^. These shape variables were used in all the subsequent morphometric comparisons.

### Statistical analysis

#### Principal components analysis

Principal Component Analysis (PCA) is an ordination method that enables the reduction of dimensionality for a given dataset and summarizing the variation within the data^96^. This is achieved by performing a singular value decomposition of the data matrix to extract eigenvectors that constitute the principal components. Each axis explains the percentage of variance described by the corresponding eigenvalues^97^. In this case, we performed a PCA to assess the morphological affinities between the individual from Toca dos Coqueiros and other populations from the region. In order to visualize population distribution in shape space, we plotted the obtained PC scores along the axes that explain the majority of the variation in the dataset. The PCA was carried out using PAST software v. 3.20^98^.

#### Euclidean distances and Ward’s method

The Euclidean distance is the most basic metric distance, indicating the length of a segment between two points in geometric space. We calculated Euclidean distances among all the samples based on the sample mean for the ten linear cranial measurements. Those distances, which are useful to assess shape similarities among early and late Holocene groups were used for estimating Ward’s clusters. Ward's clustering method of minimum variance^99^ constitutes a procedure that allows the estimation of hierarchical groups from subsets containing individuals that present similarities to one another. For each sample, a recursive algorithm is calculated to join two clusters with the smallest increase in the total value of the sum of the squares of the differences within each cluster, in relation to the centroid of the cluster. The initial distances of the cluster in Ward's method of minimum variance are defined as the square of the Euclidean distance between samples. These analyses were performed with PAST software v. 3.20^98^.

#### Discriminant analysis

The Discriminant Function Analysis (DFA) is a statistical procedure that finds linear combinations of the original variables that best indicate the differences between known groups in contrast to the variable variances within the groups. It is commonly used to classify unknown individuals into pre-assigned groups, assuming a normal distribution for the trait(s)^100^. In this case, we used the previously obtained PC scores to perform two DFA to assess the biological sex of the individual from Toca dos Coqueiros. First, we included all individuals in the comparative sample (88 males, 20 females). Afterwards, we performed a second DFA including only the Lagoa Santa series (27 males, 12 females). Besides providing a sample with a more balanced distribution of both sexes, this population exhibits a morphology that has been described as more similar to that of the Toca dos Coqueiros individual. In both cases, equal group probability was assumed and both analyses were also performed with a leave-one-out cross-validation procedure. All DFA were carried out using PAST software v. 3.20^98^.

## Supplementary Information

Supplementary Information.
